# Effect of Manitoba-Grown Red-Osier Dogwood Extracts on Recovering Caco-2 Cells from H_2_O_2_-Induced Oxidative Damage

**DOI:** 10.3390/antiox8080250

**Published:** 2019-07-28

**Authors:** Runqiang Yang, Qianru Hui, Qian Jiang, Shangxi Liu, Hua Zhang, Jiandong Wu, Francis Lin, Karmin O, Chengbo Yang

**Affiliations:** 1 College of Food Science and Technology, Nanjing Agricultural University, Nanjing 210095, China; 2 Department of Animal Science, University of Manitoba, Winnipeg, MB R3T 2N2, Canada; 3 Guelph Research & Development Centre, Agriculture and Agri-Food Canada, 93 Stone Road West, Guelph, ON N1G 5C9, Canada; 4 Department of Physics, University of Manitoba, Winnipeg, MB R3T 2N2, Canada; 5 St. Boniface Hospital Research Centre, Winnipeg, MB R2H 2A6, Canada

**Keywords:** red-osier dogwood, antioxidative effect, H_2_O_2_, transepithelial resistance (TEER), Caco-2 cells

## Abstract

Red-osier dogwood, a native species of flowering plant in North America, has been reported to have anti-oxidative properties because of abundant phenolic compounds; this could be promising as a functional food or a feed additive. In the present study, an oxidative damage model using 1.0 mM hydrogen peroxide (H_2_O_2_) in Caco-2 cells was established to evaluate the antioxidative effects of red-osier dogwood extracts (RDE). The results showed that 1.0 mM H_2_O_2_ pre-exposure for 3 h significantly decreased cell viability, and increased interleukin 8 (IL-8) secretion and the intracellular reactive oxygen species (ROS) level. Caco-2 cells were treated with 100 µg/mL RDE for 24 h after pre-exposure to H_2_O_2_. It was found that the decreased cell viability caused by H_2_O_2_ was significantly restored by a subsequent 100 µg/mL RDE treatment. Furthermore, the IL-8 secretion and ROS level were significantly blocked by RDE, accompanied by the enhanced gene expression of hemeoxygenase-1 (HO-1), superoxide dismutase (SOD), and glutathione peroxidase (GSH-Px), and the enhanced protein expression of the nuclear factor (erythroid-derived 2)-like 2 (Nrf-2). Moreover, RDE improved barrier functions in Caco-2 cells. Using RDE reduced the diffusion of fluorescein isothiocyanate (FITC)-dextran and increased the transepithelial resistance (TEER) value. The relative mRNA level of tight junction claudin-1, claudin-3, and occludin was elevated by RDE. These extracts also repaired the integrity of zonula occludens-1 (ZO-1) damaged by H_2_O_2_ and increased the protein expressions of ZO-1 and claudin-3 in the H_2_O_2_-pretreated cells. These results illustrated that RDE reduced the ROS level and enhanced the barrier function in oxidative-damaged epithelial cells.

## 1. Introduction

Phytochemicals, also referred to as phytobiotics or phytogenics, are natural bioactive products derived from plants. They include mainly essential oils [1], flavonoids [2], terpenes [3], polysaccharides [4], and phenolic acid [5]. Currently, phytochemicals were studied widely because of their multiple functions in human and animal health [6,7]. Phytochemicals have broad spectrum functions that are antioxidative [8], anti-microbial [9], anti-inflammatory [10], and anticancer in nature [11]. Research has investigated the profile of phenolic compounds and antioxidant activity of finger millet varieties [12,13]. Twenty phenolic compounds were identified including 18 flavonoids with the predominant flavonoids of catechin and epicatechin. Chen et al. [14] revealed that the dominant phytochemicals in wheat were phenolic acids with high antioxidative capacity in vitro. In addition, the beneficial functions of soy isoflavone [15,16] and lutein [8] have drawn extensive attention recently. Burgeoning evidence indicates that phytochemicals extracted from the plants could be a good source of functional foods or potential additives for functional foods and animal feeds.

Red-osier dogwood (*Cornus stolonifera* Michx.) is native to North America and is commonly found in marshes, low meadows, and along river banks from Labrador to Alaska [17]. In Canada, it is a precious plant resource and rich in phenolic components, including anthocyanins with strong antioxidative activity [18]. At present, ground red-osier dogwood has been investigated in animal feeds to improve growth performance, feed intake, disease prevention, and meat quality [19,20]. Researchers [19,20] investigated the effects of ground red-osier dogwood supplementation on the digestibility, blood metabolites, and acute phase response in beef heifers. The results showed that ground red-osier dogwood could increase the feed digestibility and plasma concentration of haptoglobin and serum amyloid A in a ground red-osier dogwood dose-dependence. Dietary 4% ground red-osier dogwood supplementation attenuated oxidative stress and improved the growth performance of the *E. coli* infected weaned piglets, as revealed by Koo et al. [21,22] and Jayaraman et al. [21,22]. The beneficial effects of ground red-osier dogwood on animal growth and feed digestion might be associated with the oxidative homeostasis of the intestinal cells and the integrity of the intestinal barrier that are maintained by the antioxidative function of phytochemicals in the ground red-osier dogwood.

Cellular oxidative and inflammatory models have been widely used to investigate the potential mechanisms of phytochemicals involved in human and animal health. The intestinal porcine epithelial cell line J2 (IPEC-J2) and human colon adenocarcinoma cell line-2 (Caco-2) provide useful cell models for investigating the antioxidant mechanism of phytochemicals in the intestine [8,21,22]. Omonijo et al. [10] revealed the anti-inflammatory mechanism of thymol in the inflammatory models of lipopolysaccharide (LPS)-induced IPEC-J2 cells. Yang et al. [23] investigated the effects of resveratrol on the oxidative model of IPEC-J2 cells, and found that resveratrol protects IPEC-J2 cells from oxidative damage by stimulating the Nrf-2 pathway. Wu et al. [24] revealed that phytochemicals increase the expressions of tight junction proteins including occludin, zona occludens (ZO-1), and induce the upregulation of antioxidant enzymes including superoxidedismutase (SOD), hemeoxygenase-1 (HO-1), catalase (CAT), and glutathione peroxidase (GSH-Px) in the oxidative model of Caco-2 cells. A variety of adverse factors could cause the production of reactive oxygen species (ROS), resulting in oxidative damage of the intestinal barrier. Moreover, persistent oxidative damage could trigger the development of the intestinal diseases, including colon cancer [25]. Therefore, the intake of natural phytochemicals could maintain the health of the gut and be considered an effective way to reduce the occurrence of intestinal diseases. Although ground red-osier dogwood has been investigated as potential animal feed additives to improve animal performance and meat quality, the mechanisms of ground red-osier dogwood in maintaining the oxidative homeostasis and the integrity of the intestinal barrier are still not fully understood.

In the present study, it is hypothesized that red-osier dogwood extracts (RDE) act as antioxidant agents capable of exerting protective effects against chemical-induced damage in intestinal epithelial cells. Therefore, an oxidative damage model of Caco-2 cells induced by hydrogen peroxide (H_2_O_2_) was established, and the mechanisms of RDE in recovering the intestinal cells from oxidative damage were investigated by examining the antioxidant system and the tight junction protein expression. 

## 2. Materials and Methods

### 2.1. Materials and Reagents

RDE powder (moisture 7.3%; total phenolic content 26.51 gallic acid equivalent (GAE)/100 g including gallic acid 1670.09 mg/100 g and rutin 2745.49 mg/100 g) from leaves was provided by Red Dog Enterprises Ltd. (Swan River, MB, Canada). Hydrogen peroxide (H_2_O_2_), 2′,7′dichlorofluorescein diacetate (DCFH-DA), 4 kDa fluorescein isothiocyanate-dextran (FD4) and paraformaldehyde (PFA) were purchased from Sigma-Aldrich (Oakville, ON, Canada). Hanks’ balanced salt solution (HBSS), DMEM/F12 medium, trypsin (0.25%), anti-anti (penicillin (100 IU/mL), streptomycin (100 μg/mL), and 0.25 μg/mL of amphotericin B) and fetal bovine serum (FBS) were purchased from Invitrogen (Fisher Scientific, Ottawa, ON, Canada). T-25 or T-75 culture flasks, Transwell permeable supports (0.4 μm pore size), and cell culture plates were purchased from Corning (Fisher Scientific, Ottawa, ON, Canada).

### 2.2. Cell Culture

Caco-2 human colon cancer cell was purchased from the American Type Culture Collection (ATCC, Manassas, VA, USA). Cells were grown in DMEM-Ham’s F-12 (1:1) supplemented with 10% FBS and 1% anti-anti, and maintained in an atmosphere of 5% CO_2_ at 37 °C. Cells were cultured in a T-25 or T-75 culture flask with a medium change every two days until 90% confluence. Then, they were digested with 0.25% trypsin (at 37 °C for 1–2 min) and seeded in a cell culture plate (96-, 24-, 12-, or 6-well) to culture for further experiments.

### 2.3. Experiment Design

Cytotoxicity of H_2_O_2_ (ranges from 0.2 to 2.0 mM) on Caco-2 cells was tested by a viability assay, and the optimal concentration of H_2_O_2_ was chosen for inducing oxidative damage of Caco-2 cells. Cells were treated with 1 mM of H_2_O_2_ for 3 h firstly, and then washed with PBS (a pre-warmed at 37 °C water bath) one time. Afterward, the cells treated with 100 μg/mL RDE for 24 h were considered the treatment group (T) according to the results. The cells treated with H_2_O_2_ and followed with a standard medium [DMEM-Ham’s F-12 (1:1) supplemented with 10% FBS and 1% anti-anti] for 24 h were defined as treatment control (TC). The cells without H_2_O_2_ and RDE treatment were negative control (NC). In brief, the H_2_O_2_ work solution was directly diluted using a medium from 10 M H_2_O_2_. The RDE solution was prepared according to the required concentrations using a medium.

### 2.4. Cell Viability Assay

After treatments, cell viability was measured using the water-soluble tetrazolium salts (WST-1) Cell Proliferation Reagent (Sigma Aldrich, Roche, Indianapolis, IN, USA) according to the manufacturer’s instructions. Briefly, Caco-2 cells were seeded into 96-well plates at a density of 2 × 10^4^ cells/well and cultured in a complete medium for about 5 days (90% confluent). After the different treatments (NC, TC, and T), cells were washed one time with PBS and then treated with 100 μL fresh medium containing 10% WTS-1 and incubated for 1.5 h at 37 °C in an atmosphere of 5% CO_2_. The absorbance at 450 nm was measured using a Synergy™ H4 Hybrid Multi-Mode Microplate Reader (BioTek, Winooski, VT, USA). Cell viability was presented as a percentage of control cells.

### 2.5. IL-8 Determination

IL-8 was determined using IL-8 Human Uncoated ELISA Kit (cat. #88-8086, Invitrogen, Carlsbad, CA, USA). Cells were cultured in a 12-well plate with the inoculum density of 2 × 10^5^ cells/well and treated with H_2_O_2_ and RDE as described above. After, 100 μL of the medium was used to determine IL-8 content according to the kit instructions.

### 2.6. Reactive Oxygen Species (ROS) Assay

Cellular ROS was measured using a BD FACSCalibur flow cytometer (BD Biosciences, San Jose, CA, USA) [26]. Cells were cultured in a T-25 flask with a inoculum density of 1 × 10^6^ cells/well until 90% confluence reached. They were treated with H_2_O_2_ and RDE as described above. After treatments, cells were washed with PBS two times and treated with 1 mL DCFH-DA solution (10 µM in PBS) for 30 min at 37 °C. Then, cells were washed with PBS two times and fixed with 4% PFA for 15 min at room temperature. After that, they were digested with 0.25% trypsin (at 37 °C) to monoplast, washed with PBS, and then centrifuged to collect cells for flow cytometry analysis. A total of 10,000 cells was calculated, and fluorescence intensity was measured.

### 2.7. Glutathione Determination

Total glutathione (GSH) and oxidized glutathione (GSSG) were determined using a Glutathione Colorimetric Detection Kit (cat. #EIAGSHC, Invitrogen). Cells were cultured in 12-well plate with a inoculum density of 2 × 10^5^ cells/well and treated with H_2_O_2_ and RDE as described above. Afterward, cells were washed with ice-cold PBS and resuspended in 200 μL of ice-cold 5% aqueous 5-sulfo-salicylic acid dehydrate (SSA) and followed by the freeze–thaw cycling to lyse cells. After, lysed cells were centrifuged at 14,800× *g* at 4 °C for 15 min. The dilution and assay were conducted by following the kit instructions. The reduced GSH was calculated by an equation: reduced GSH = total GSH − GSSG.

### 2.8. Transepethial Electrical Resistant (TEER) Measurement

The TEER of cell monolayers was measured using a Millicell Electrical Resistance System (ESR-2) (Millipore-Sigma) according to our previously published procedure [10]. Caco-2 cells were seeded onto Transwells (Polyester membrane, 12 wells, 12 mm diameter inserts, Corning Costar, Fisher Scientific) at a density of 1 × 10^5^ cells/insert. The TEER value was monitored every other day. When a monolayer of cells was completely differentiated (about 10 days), cells were treated with H_2_O_2_ and RDE, and TEER values were measured after H_2_O_2_ treatment for 6 h and after RED treatment for 24 h, respectively. The data were presented as a percentage of initial value before treatments.

### 2.9. Measurement of Cell Permeability

To quantify the paracellular permeability of cell monolayers, 1 mg/mL of FD4 (46944-500MG-F, Sigma-Aldrich) was added to the apical side of the inserts (Polyester membrane, 12 wells, 12 mm diameter inserts, Corning Costar, Fisher Scientific, Ottawa, ON, Canada). The cell culture and the treatments were the same as that of TEER measurement. The basolateral medium aliquots were taken after 2 h of incubation at 37 °C in an atmosphere of 5% CO_2_. Then, 100 μL of the medium was transferred into 96-well plates and the diffused fluorescent tracer was then measured by fluorometry (excitation, 485 nm; emission, 528 nm) using a Synergy™ H4 Hybrid Multi-Mode Microplate Reader (BioTek, Winooski, VT, USA) [10].

### 2.10. RNA Extraction and Real-Time PCR

Total RNA isolation and real-time PCR were conducted according to Omonijo et al. [10]. The reference gene was GAPDH (glyceraldehyde-3-phosphate dehydrogenase). The primers for real-time PCR analysis were designed with the Primer-Blast based on the published cDNA sequence in the Gene Bank (https://www.ncbi.nlm.nih.gov/tools/primer-blast/). The information of genes detected and the primers are shown in Table 1.

### 2.11. Immunofluorescent Staining

Cells with a inoculum density of 1 × 10^5^ cells/well were cultured onto coverslips (24-well plate, Fisher Scientific) pre-coated with collagen and fixed with 4% PFA after treatments as described above. The cells were washed with PBS 2 times and incubated with 0.3% Triton X-100 in PBS (PBS/Triton) at room temperature for 10 min. For β-actin staining, the treated cells were incubated with Phalloidin, CF488A (1:100 dilution in PBS, Biotium, Inc., Fremont, CA, USA) at room temperature for 1 h. The cells were then washed three times with PBS and mounted with Vectashield mounting medium with DAPI (Vector Laboratories, Inc., Burlingame, CA, USA). For ZO-1 staining, cells were blocked with 5% goat serum (Jackson ImmunoResearch Laboratories, West Grove, PA, USA) for 1 h and then incubated with an anti-rabbit ZO-1 polyclonal antibody (cat. # 61-7300, 1:100 dilution, Thermal Scientific, Ottawa, Canada) at 4 °C overnight. The cells were then washed three times with PBS and incubated with an Alexa fluor 488 goat anti-rabbit antibody (Thermal Scientific, cat. # A-11034) for 1 h at room temperature. Rinsed cells were mounted with Vectashield Mounting Medium with DAPI (Vector Laboratories, Inc.)**.** The images were taken by a Zeiss Fluorescence Microscope (Carl Zeiss Ltd., Toronto, ON, Canada) [10].

### 2.12. Western Blotting

Cells were cultured in a 6-well plate with a inoculum density of 4 × 10^5^ cells/well and total protein was extracted after the treatments described above. Protein extraction was conducted using a total protein extraction kit according to instructions provided by the manufacturer (Thermo Scientific, Ottawa, ON, Canada). A portion of protein was quantified using a BCA protein assay kit (Thermo Scientific). Then proteins were heat-denatured in an SDS-PAGE loading buffer. Proteins were electrophoresed on polyacrylamide gels and electro-transferred to mini-size nitrocellulose membranes (Bio-Rad, Laboratories Ltd., Montreal, QC, Canada). The immunoreaction was achieved by incubation of the membranes, previously blocked with 5% non-fat dry milk in Tris-buffered saline with 0.1% of Tween 20 (TBST), followed by incubation with rabbit anti-Nrf-2 (Thermal Scientific, cat. #PA5-68817), rabbit anti-ZO-1 (Thermal Scientific, cat. #61-7300), rabbit anti-claudin-1(Thermal Scientific, cat. #34-1700), rabbit anti-claudin-3 (Thermal Scientific, cat. #51-9000), and rabbit anti-occludin (Thermal Scientific, cat. #71-1500) proteins after being diluted (1:1000, Abcam Inc., Toronto, ON, Canada) overnight at 4°C. Afterward, it was washed five times with TBST. Detection of the immune complexes was performed with a horseradish peroxidase-conjugated secondary antibody (1:5000, goat anti-rabbit, Jackson ImmunoResearch Laboratories), then the membrane was washed 5 times, 5 min per time. The Clarity^TM^ Western ECL Substrate was applied to the blot according to the manufacturer’s recommendations (Bio-Rad Laboratories Ltd.). The chemiluminescent signals were captured using a ChemiDoc MP imaging system (Bio-Rad Laboratories Ltd.), and Image Lab 6.0 was used to quantify the intensity of the bands (Bio-Rad Laboratories Ltd.). β-actin (from mouse, Thermal Scientific, cat. #AM4302) was set as the internal reference.

### 2.13. Statistical Analysis

Data were presented as means ± standard deviations (SD). Statistical analysis was performed using GraphPad Prism 7 (GraphPad Software, La Jolla, CA, USA). Differences between the means were evaluated by one-way ANOVA. Duncan’s multiple range test was used. Level of significance was set at *P* < 0.05.

## 3. Results

**Figure 1:**
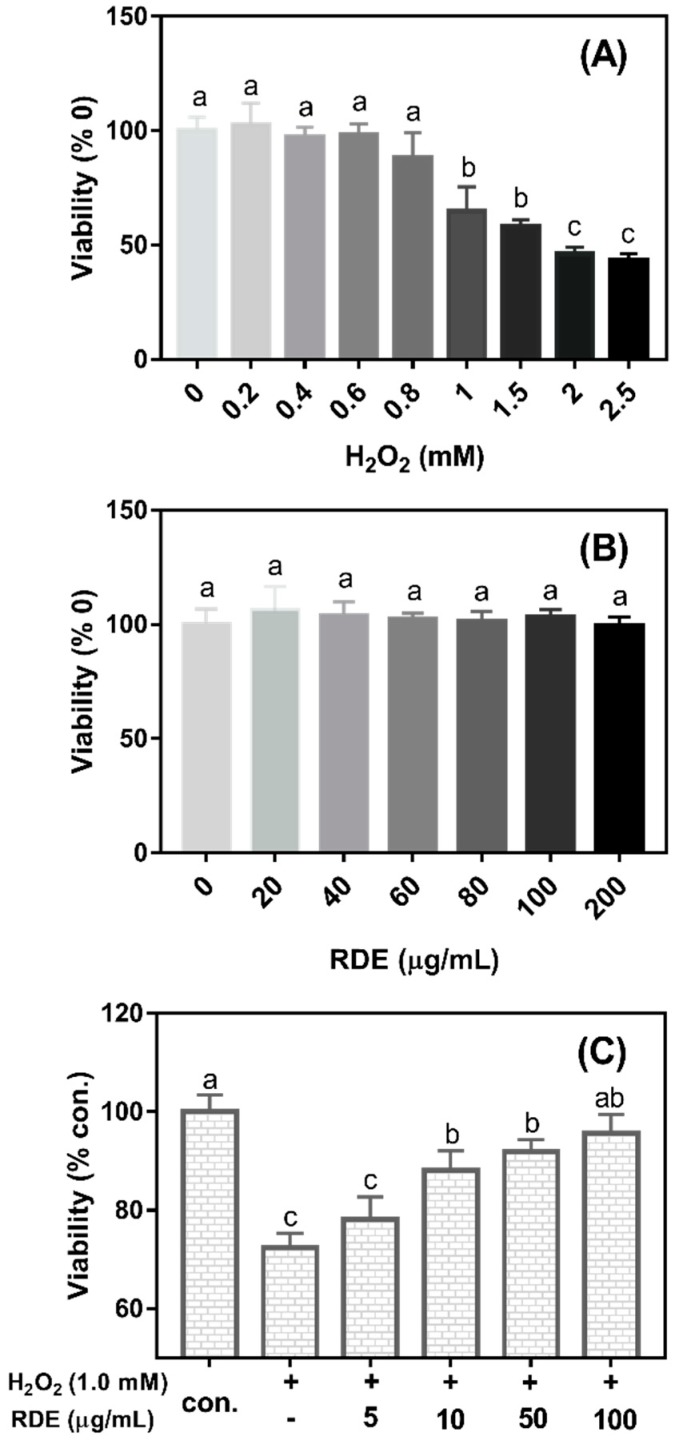
Effect of H_2_O_2_ and red-osier dogwood extracts (RDE) on the cell viability of Caco-2 cells. (**A**) Cells were treated with different concentrations of H_2_O_2_ for 24 h, and then cell viability was detected. (**B**) Cells were treated with RDE for 24 h, and then cell viability was detected. (**C**) Cells were treated with 1.0 mM H_2_O_2_ for 3 h, and then treated with a medium containing different RDE concentrations for 24 h. Cells were seeded in a 96 well plate at a density of 2 × 10^4^/well and cultured for 5 d. Cell viability was expressed as a percentage of control (without H_2_O_2_ and RDE). The data were presented as mean ± SD, *n* = 5. Different lower case letters indicate a significant difference at *P* < 0.05.

As shown in Figure 1A, the cell viability significantly decreased when the concentrations of H_2_O_2_ were more than 0.8 mM (*P* < 0.05). The cell viability was reduced at 35.15% when 1 mM H_2_O_2_ was added in the medium compared with the control (0 mM H_2_O_2_). Therefore, 1 mM H_2_O_2_ was selected to induce oxidative damage in this study. As shown in Figure 1B, the cell viability was not changed when cells were treated with the RDE at the concentrations of 0–200 μg/mL (*P* > 0.05). As shown in Figure 1C, when cells were pre-treated with 1 mM H_2_O_2_ for 3 h and then incubated with different concentrations of RDE for 24 h, RDE showed a dose-dependent recovery of the cell viability that was compromised by 1 mM H_2_O_2_ and the cells treated with 100 μg/mL of RDE had similar viability with the control cells (*P* > 0.05). Therefore, 100 μg/mL of RDE was used in the subsequent experiments.

**Figure 2:**
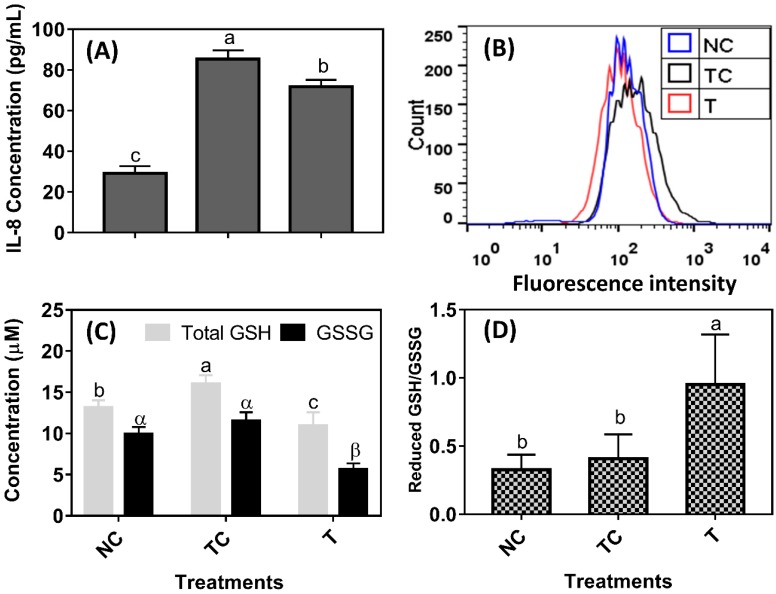
The effect of red-osier dogwood extracts (RDE) on interleukin (IL)-8 secretion (**A**), reactive oxygen species (ROS) level (**B**), glutathione (GSH) content (**C**), and reduced GSH/oxidized GSH (GSSG) (**D**) of Caco-2 cells after H_2_O_2_ treatment. NC indicates negative control: cells were cultured with a medium without H_2_O_2_ and RDE for 27 h (3 h + 24 h). TC indicates treatment control: cells were cultured with a medium containing 1 mM of H_2_O_2_ for 3 h, and then treated with a medium for 24 h. T indicates RDE treatment: cells were cultured with a medium containing 1 mM of H_2_O_2_ for 3 h, and then treated with a medium containing 100 μg/mL of RDE for 24 h. The data were presented as mean ± SD, *n* = 3. Different lower case letters and Greek letters indicate a significant difference at *P* < 0.05.

H_2_O_2_ treatment induced a significant amount of IL-8 secretion and they were increased almost two-fold when compared with the control cells (NC) (*P* < 0.05) (Figure 2A). The 100 μg/mL of RDE reduced IL-8 secretion in the cells when compared with the treatment control cells (TC) (*P* < 0.05). However, IL-8 secretion was still higher in the cells treated with RDE (T) than in the control cells (NC) (*P* < 0.05). As shown in Figure 2B, the intracellular fluorescent intensity of cells treated with H_2_O_2_ (TC) was higher than those of the control cells (NC) and the incubation of H_2_O_2_-pretreated cells with RDE for 24 h reduced the ROS to the level of control. The H_2_O_2_ treatment increased total GSH content (*P* < 0.05) while the reduced GSH/ GSSS ratio remained the same (*P* > 0.05) when compared with the control cells (NC). The RDE treatment reduced both total GSH and GSSG (*P* < 0.05, Figure 2C) and had a higher reduced GSH/GSSG ratio when compared with both the NC and TC groups (*P* < 0.05, Figure 2D).

**Figure 3:**
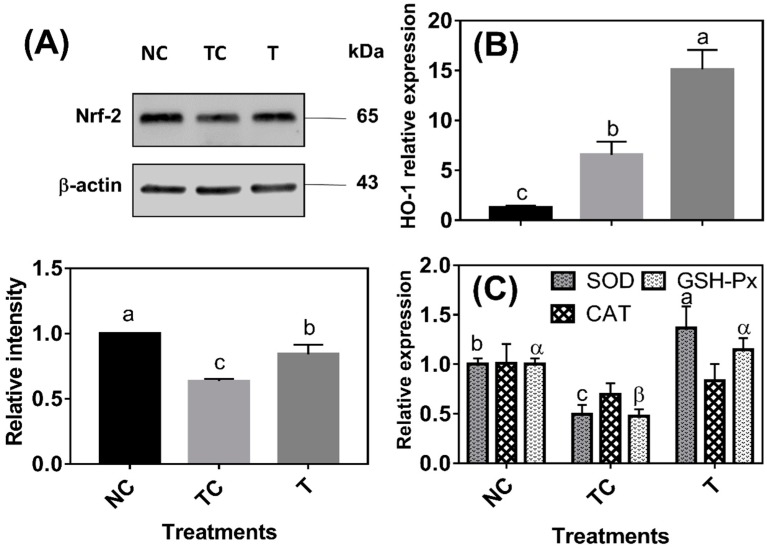
The effect of red-osier dogwood extracts (RDE) on the Nrf-2 protein level (**A**), HO-1 mRNA expression (**B**), and antioxidative enzymes mRNA levels (**C**) of Caco-2 cells after H_2_O_2_ treatment. NC indicates negative control: cells were cultured with a medium without H_2_O_2_ and RDE for 27 h (3 h + 24 h). TC indicates treatment control: cells were cultured with a medium containing 1 mM of H_2_O_2_ for 3 h, and then treated with a normal medium for 24 h. T indicates RDE treatment: cells were cultured with medium containing 1 mM of H_2_O_2_ for 3 h, and then treated with a medium containing100 μg/mL of RDE for 24 h. The data were presented as mean ± SD, *n* = 4. Different lower case letters and Greek letters indicate a significant difference at *P* < 0.05. Nrf-2: nuclear factor erythroid 2-related factor 2, SOD: superoxide dismutase; HO-1: hemeoxygenase-1; CAT: catalase; GSH-Px: glutathione peroxidase.

As shown in Figure 3A, the Nrf-2 protein abundance was lower (*P* < 0.05) in the cells treated with H_2_O_2_ (TC) than in the control cells (NC). The Nrf-2 protein abundance was higher (*P* < 0.05) in the cells treated with RDE (T) than in the cells treated with H_2_O_2_ (TC) but still lower (*P* < 0.05) than in the control cells (NC). HO-1 mRNA abundance was increased (*P* < 0.05) by 5.29 folds under H_2_O_2_ treatment compared with that of the control cells (NC) and HO-1 mRNA abundance was the highest (*P* < 0.05) in the cells treated with RDE (Figure 3B). H_2_O_2_ treatment significantly decreased the mRNA levels of SOD and GSH-Px, and the mRNA levels of the two enzymes were increased by followed RDE treatment (*P* < 0.05). However, there was no significant difference (*P* > 0.05) observed in the mRNA level of CAT among the three treatment groups (Figure 3C).

**Figure 4:**
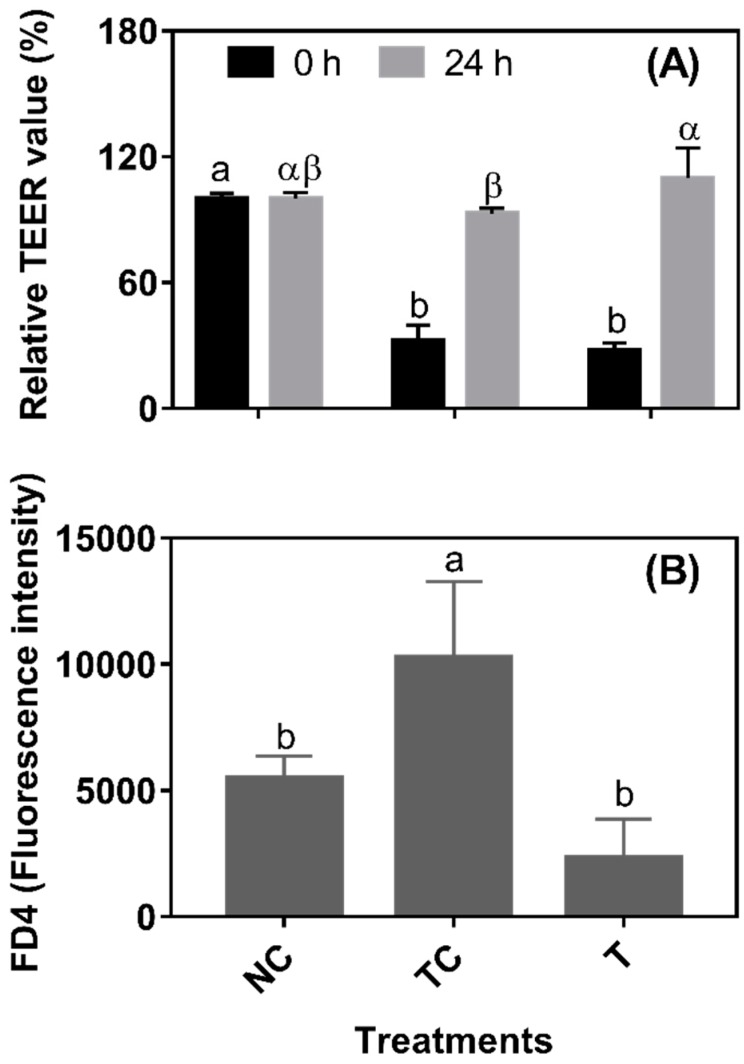
The effect of red-osier dogwood extracts (RDE) on the transepithelial resistance (TEER) (**A**) and permeability (**B**) of Caco-2 cells after H_2_O_2_ treatment. NC indicates negative control: cells were cultured with DMEM solution without H_2_O_2_ and RDE for 27 h (3 h + 24 h). TC indicates treatment control: cells were cultured with a medium containing 1 mM of H_2_O_2_ for 3 h, and then treated with a medium for 24 h. T indicates RDE treatment: cells were cultured with a medium containing 1 mM of H_2_O_2_ for 3 h, and then treated with medium containing 100 μg/mL of RDE for 24 h. TEER was measured after H_2_O_2_ treatment for 3 h and after RDE treatment for 24 h, respectively. The data were presented as mean ± SD, *n* = 4. Different lower case letters and Greek letters indicate a significant difference at *P* < 0.05.

**Figure 5:**
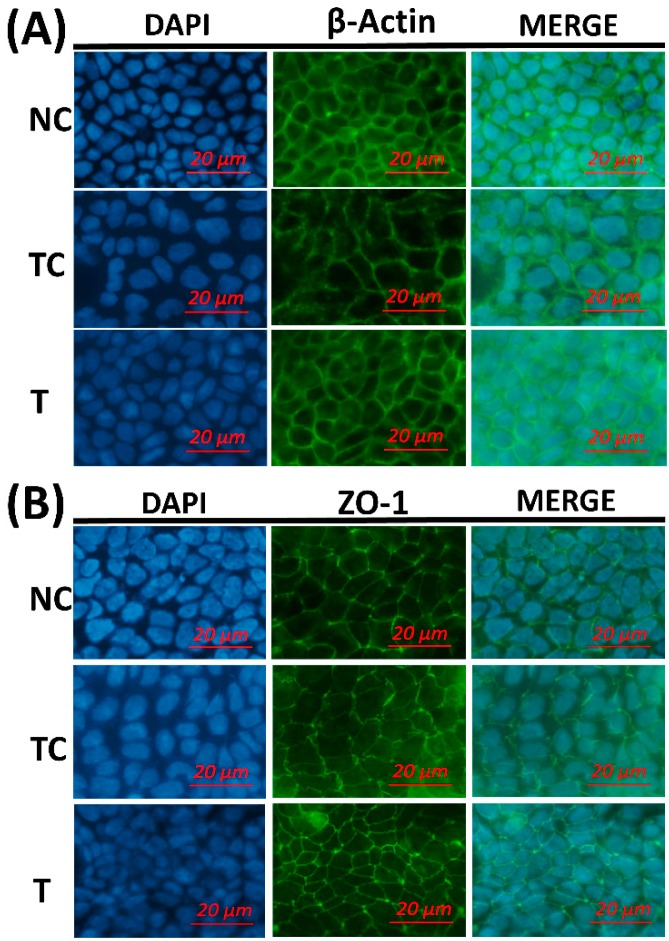
The effect of red-osier dogwood extracts (RDE) on the morphological changes of β-actin fiber and tight junction in Caco-2 cells after H_2_O_2_ treatment. (**A**) The morphological changes of β-actin and (**B**) The morphological changes of ZO-1. NC indicates negative control: cells were cultured with a medium without H_2_O_2_ and RDE for 27 h (3 h + 24 h). TC indicates treatment control: cells were cultured with a medium containing 1 mM of H_2_O_2_ for 3 h, and then treated with a medium for 24 h. T indicates RDE treatment: cells were cultured with a medium containing 1 mM of H_2_O_2_ for 3 h, and then treated with medium containing 100 μg/mL of RDE for 24 h. ZO-1: zonula occludens-1.

In Figure 4A, after a 3 h H_2_O_2_ treatment, the TEER values were significantly decreased (set as 0 h from RDE treatment) (*P* < 0.05). There was no significant difference (*P* > 0.05) observed in the TEER values between the TC and NC groups and between the NC and T groups when cells were further cultured for 24 h. However, the TEER value was higher (*P* < 0.05) in the cells treated with RDE (T) than in the treatment control cells (TC). As shown in Figure 4B, the leakage of FD4 was higher (*P* < 0.05) in the TC group than in the NC and T groups. However, there was no significant difference (*P* > 0.05) in the leakage of FD4 observed between the NC and T groups. As shown in Figure 5, the cytoskeletal structure of the β-actin fiber was partially disorganized in the TC group by H_2_O_2_ treatment, while incubation with RDE after H_2_O_2_ treatment could alleviate the damage induced by H_2_O_2_. The morphology of tight junction had similar changes as cytoskeleton after different treatments.

**Figure 6:**
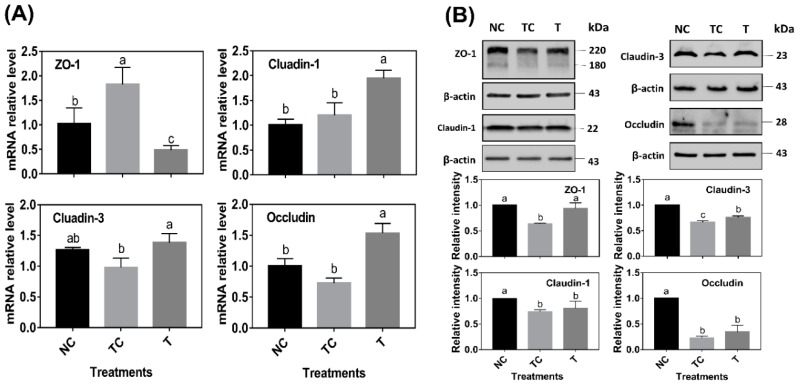
The effect of red-osier dogwood extracts (RDE) on the tight junction mRNA abundance (**A**) and protein abundance (**B**) of Caco-2 cells after H_2_O_2_ treatment. NC indicates negative control: cells were cultured with a medium without H_2_O_2_ and RDE for 27 h (3 h + 24 h). TC means treatment control: cells were cultured with a medium containing 1 mM of H_2_O_2_ for 3 h, and then treated with a medium for 24 h. T indicates RDE treatment: cells were cultured with a medium containing 1 mM of H_2_O_2_ for 3 h, and then treated with a medium containing 100 μg/mL of RDE for 24 h. The data were presented as mean ± SD, *n* = 3. Different lower case letters indicate a significant difference at *P* < 0.05. ZO-1: zonula occludens-1. The black bar, gray bar, and light black bar indicated the treatments NC, TC, and T, respectively.

As shown in Figure 6A, ZO-1 mRNA abundance was significantly increased by H_2_O_2_ treatment (*P* < 0.05) while there was no difference (*P* > 0.05) observed in the mRNA abundance of claudin-1, claudin-3, and occludin when compared with the control cells (NC). However, the cells treated with RDE had a higher level of claudin-1, claudin-3, and occludin mRNA abundance and a lower level of ZO-1 mRNA abundance than those in the other two treatments (*P* < 0.05). As shown in Figure 6B, H_2_O_2_ treatment (TC) significantly decreased the protein abundance of ZO-1, claudin-1, claudin-3, and occludin (*P* < 0.05) when compared with the control cells (NC). RDE treatment (T) significantly increased the protein abundance of ZO-1 and claudin-3 (*P* < 0.05) when compared with the TC group.

## 4. Discussion

Numerous studies have shown that the occurrences of the intestinal disease are associated with a defective barrier function that was caused by oxidative damage [27]. Therefore, controlling oxidative damage and repairing the intestinal barrier could be effective ways to prevent many intestinal diseases. Phytochemicals have been reported to reduce the production of cellular ROS [28] and the incidence of intestinal inflammation [10]. The supplementation of ground red-osier dogwood in feeds decreased the serum malondialdehyde content and increased the SOD levels in the *Escherichia coli* F4 K88^+^ infected animals, as reported by a previous study [21]. In the present study, an oxidative damage model was established by using H_2_O_2_ in Caco-2 cells, aiming to evaluate the therapeutic effects of RDE on intestinal oxidative damage.

To establish an H_2_O_2_-induced oxidative injury model with Caco-2 cells, the dosage effects of H_2_O_2_ on cell viability was investigated firstly. The results showed that the H_2_O_2_ treatment significantly reduced cell viability at more than 0.8 mM, indicating that a high dosage of H_2_O_2_ inhibited the growth of the cells or was toxic to the cells. These findings are consistent with previous studies of H_2_O_2_ and Caco-2 cells [29]. However, a wide concentration range of RDE (up to 200 μg/mL, equal to 53 μg/mL total phenolic compounds) did not show a negative effect on cell viability, suggesting that higher RDE containing higher phenolic compounds might have better pharmaceutical effectiveness. Moreover, our results demonstrated that 100 μg/mL of RDE completely recovered the cell viability that was compromised by 1 mM H_2_O_2_. Therefore, our results suggest that RDE exerts protective effects against H_2_O_2_-induced oxidative damage in intestinal epithelial cells in vitro.

H_2_O_2_-induced oxidative stress triggered an imbalanced redox state and excessive ROS accumulation in Caco-2 cells, which in turn induced inflammatory responses evidenced by an increased IL-8 level. IL-8, a chemokine that can be produced by epithelial cells, has been recognized as an indicator of the inflammatory response [30]. The mediators can activate signal transduction cascades as well as inducing variations in transcription factors, such as Nrf-2, which mediate immediate cellular stress responses [31]. The oxidative stress and inflammatory responses together exacerbated the damage of both cellular structure and biomacromolecules. After a 3 h treatment with H_2_O_2_ and following a 24 h treatment with 100 μg/mL of RDE, the viability of Caco-2 cells was significantly restored, accompanied by decreased levels of IL-8 and ROS, indicating the inflammatory responses and oxidative damage were alleviated. The anti-oxidative components in RDE including rutin and phenolic acids are highly effective at scavenging oxygen radicals [32].

A recent study shows that rutin attenuates inflammatory responses induced by lipopolysaccharide in an in vitro mouse muscle cell (C2C12) model and supplementation of rutin or rutin-containing plant extracts may hold promising potential for attenuating oxidative stress and inflammation [33]. In addition, RDE increased the ratio of reduced GSH/GSSG, and reduced the consumption of reduced GSH. The results indicate that RDE can alleviate the occurrence of inflammation and oxidative damage by scavenging ROS production directly.

To further investigate the therapeutic mechanism of RDE on H_2_O_2_-induced oxidative damage, the protein expression of Nrf-2 and gene expression of responsive gene HO-1 and antioxidative enzyme SOD, CAT, and GSH-Px were measured. The protein expression of Nrf-2 was induced (Figure 3A) and the mRNA expression of HO-1 was significantly upregulated after the addition of RDE (Figure 3B). In addition, the mRNA abundance of SOD and GSH-Px also showed the same trend as HO-1 (Figure 3C). This study revealed that RDE activated Nrf-2, mediated the antioxidant gene expression, and enhanced the antioxidant capacity of cells, thereby preventing the inflammation response and oxidative damage.

The intact gut barrier is important to prevent cell damage and restore cellular function caused by intestinal inflammation [10] and oxidative stress [34]. The TEER and permeability, as measured by FD4 diffusion, were often used to evaluate the integrity and tightness of the barrier formed by epithelial cells. Caco-2 cells were measured until the stable TEER formed by the tight junction. The permeability of the barrier after the 24 h treatment was also investigated by measuring the fluorescence intensity of FITC-Dextran (FD4) in the basolateral side diffused from the apical side. The H_2_O_2_ treatment lowered the TEER value and increased the permeability, suggesting that H_2_O_2_ treatment led to the increase in cell permeability [35], and resulted in a defective barrier function of cells. However, TEER values were increased and permeability was decreased by RDE treatment for 24 h, indicating that the barrier function of Caco-2 cells was improved significantly. A recent study showed that intact epithelial barrier functions are necessary in order to maintain the homeostatic state in response to physiological host-gut microbiome cross-talks, and therefore the maintenance of epithelial barrier function is crucial for alleviating gut inflammation [36]. It has been reported that phytochemicals extracted from apples can increase the TEER value of Caco-2 cells [37], and this might be attributed to the antioxidative effects of phytochemicals.

The morphology of the cytoskeleton and tight junction was visualized by β-actin and ZO-1 immunofluorescent staining. H_2_O_2_ treatment disorganized the β-actin fibrin and diffused cell tight junction protein ZO-1. This indicates that H_2_O_2_ caused oxidative damage to the cell membrane monolayer. The addition of RDE obviously restored cell structure and stabilized morphological characteristics of ZO-1.

In this study, the mRNA and protein abundance of tight junction proteins, including ZO-1, claudin-1, claudin-3, and occluding, were determined. When compared with the sole H_2_O_2_ treatment group, RDE supplemented to cells enhanced the mRNA abundance of tight junction protein claudin-1, claudin-3, and occludin, but decreased the ZO-1 mRNA level in the Caco-2 cells. Firstly, this might be attributed to the fact that the sensitivity of gene expression of the tight junction proteins was different when responding to H_2_O_2_ [38]. The transcription of ZO-1 was more sensitive to H_2_O_2_, and it could rapidly respond to oxidative stress. Secondly, it may also be related to the ordinal expression of different tight junction proteins. Within 24 h after H_2_O_2_ treatment, intracellular H_2_O_2_ was still playing a role in cells, and the expression of ZO-1, which was sensitive to oxidative stress, was induced. However, when cells were treated with H_2_O_2_ for 3 h, followed by RDE incubation of cells for 24 h, ROS was partially removed by RDE due to its strong antioxidant effect. Therefore, the expression of ZO-1 was not upregulated.

Interestingly, claudin-3 and occludin could be upregulated by RDE treatment, indicating that there were some unknown components in RDE that could regulate claudin-3 and occludin gene expression. In terms of tight junction proteins expression, H_2_O_2_ treatment reduced the contents of ZO-1, claudin-1, claudin-3, and occludin significantly. This was not completely consistent with their mRNA expression results. Firstly, it can be attributed to protein expression lagging behind mRNA expression [24]. In addition, H_2_O_2_ treatment significantly disrupted the structure of tight junction proteins, resulting in a decrease in the protein content. After RDE treatment, the protein content of ZO-1 and claudin-3 increased significantly, but the protein content of claudin-1 and occludin did not increase. It was also evident from the staining results that the ZO-1 structure has also been significantly repaired to enhance the cellular barrier function and viability. Based on our observations in this study, there could be two reasons to explain why RDE enhances cellular barrier function. Firstly, RDE is rich in antioxidants [19,32] that have the ability to scavenge ROS directly and repair the structure of tight junction proteins. Moreover, RDE is rich in unknown components that activate the Nrf-2 pathway to enhance the antioxidant capacity of cells and induce the expression of tight junction proteins.

## 5. Conclusions

In conclusion, RDE enhances cell activity by directly scavenging ROS and strengthening the cellular antioxidant system, as well as by repairing tight junction proteins and inducing their expression.

## Figures and Tables

**Table 1:** Primers used in this study

Gene	Primer Sequences (5′ →3′)	Length (bp)	Access No.
ZO-1	CAACATACAGTGACGCTTCACA	105	NM_001355013.1
	CACTATTGACGTTTCCCCACTC		
Occludin	GACTATGTGGAAAGAGTTGAC	174	XM_017008914.2
	ACCGCTGCTGTAACGAG		
Claudin-1	TGGTGGTTGGCATCCTCCTG	232	NM_021101.4
	AATTCGTACCTGGCATTGACTGG		
Claudin-3	GCCACCAAGGTCGTCTACTC	101	NM_001306.3
	CCTGCGTCTGTCCCTTAGAC		
SOD	ATCCTCTATCCAGAAAACACG	250	NM_000454.4
	ACACCACAAGCCAAACGAC		
HO-1	TTTGAGGAGTTGCAGGAGC	179	NM_002133.2
	GTAAGGACCCATCGGAGAA		
CAT	TCCAAGGCAAAGGTATTTGAGCA	157	NM_001752.3
	CAACGAGATCCCAGTTACCATCTTC		
GSH-Px	CCTCTAAACCTACGAGGGAGGAA	107	NM_001329455.1
	GGGAAACTCGCCTTGGTCT		
GAPDH	GCACCGTCAAGGCTGAGAAC	142	NM_001289745.2
	ATGGTGGTGAAGACGCCAGT		

Note. ZO-1: zonula occludens-1, SOD: superoxidedismutase, HO-1: hemeoxygenase-1, CAT: catalase, GSH-Px: glutathione peroxidase, GAPDH: glyceraldehyde-3-phosphate dehydrogenase, bp: base pair.

## References

[1] María José A., Luis Miguel B., Luis A., Paulina B. (2012). The *Artemisia* L. genus: A review of bioactive essential oils. Molecules.

[2] Han B., Xin Z., Ma S., Liu W., Zhang B., Ran L., Yi L., Ren D. (2017). Comprehensive characterization and identification of antioxidants in *Folium Artemisiae Argyi* using high-resolution tandem mass spectrometry. J. Chromatogr. B.

[3] Patil S.P., Kumbhar S.T. (2018). Evaluation of terpene-rich extract of *Lantana camara* L. leaves for antimicrobial activity against mycobacteria using Resazurin Microtiter Assay (REMA). Beni-Suef Univ. J. Basic Appl. Sci..

[4] Zhang P., Ran R.K., Abdullahi A.Y., Shi X.L., Huang Y., Sun Y.X., Liu Y.Q., Yan X.X., Hang J.X., Fu Y.Q. (2018). The mitochondrial genome of Dipetalonema gracile from a squirrel monkey in China. J. Helminthol..

[5] Xiang J., Zhang M., Apea-Bah F.B., Beta T. (2019). Hydroxycinnamic acid amide (HCAA) derivatives, flavonoid C-glycosides, phenolic acids and antioxidant properties of foxtail millet. Food Chem..

[6] Lillehoj H., Liu Y., Calsamiglia S., Fernandez-Miyakawa M.E., Chi F., Cravens R.L., Oh S., Gay C.G. (2018). Phytochemicals as antibiotic alternatives to promote growth and enhance host health. Vet. Res..

[7] Hassan Y.I., Lahaye L., Gong M.M., Peng J., Gong J., Liu S., Gay C.G., Yang C. (2018). Innovative drugs, chemicals, and enzymes within the animal production chain. Vet. Res..

[8] Yang C., Fischer M., Kirby C., Liu R., Zhu H., Zhang H., Chen Y., Sun Y., Zhang L., Tsao R. (2018). Bioaccessibility, cellular uptake and transport of luteins and assessment of their antioxidant activities. Food Chem..

[9] Kim J.-S., Kwon Y.-S., Chun W.-J., Kim T.-Y., Sun J., Yu C.-Y., Kim M.-J. (2010). *Rhus verniciflua* Stokes flavonoid extracts have anti-oxidant, anti-microbial and α-glucosidase inhibitory effect. Food Chem..

[10] Omonijo F.A., Liu S., Hui Q., Zhang H., Lahaye L., Bodin J.-C., Gong J., Nyachoti M., Yang C. (2019). Thymol improves barrier function and attenuates inflammatory responses in porcine intestinal epithelial cells during lipopolysaccharide (LPS)-induced inflammation. J. Agric. Food Chem..

[11] Akhtar M.S., Swamy M.K. (2018). Anticancer Plants: Natural Products and Biotechnological Implements.

[12] Xiang J., Apea-Bah F.B., Ndolo V.U., Katundu M.C., Beta T. (2019). Profile of phenolic compounds and antioxidant activity of finger millet varieties. Food Chem..

[13] Xiang J., Li W., Ndolo V.U., Beta T. (2019). A comparative study of the phenolic compounds and in vitro antioxidant capacity of finger millets from different growing regions in Malawi. J. Cereal Sci..

[14] Chen Z., Ma Y., Yang R., Gu Z., Wang P. (2019). Effects of exogenous Ca^2+^ on phenolic accumulation and physiological changes in germinated wheat (*Triticum aestivum* L.) under UV-B radiation. Food Chem..

[15] Jeong Y.J., An C.H., Park S.-C., Pyun J.W., Lee J., Kim S.W., Kim H.-S., Kim H., Jeong J.C., Kim C.Y. (2018). Methyl jasmonate increases isoflavone production in soybean cell cultures by activating structural genes involved in isoflavonoid biosynthesis. J. Agric. Food Chem..

[16] Hsu C., Wu B.-Y., Chang Y.-C., Chang C.-F., Chiou T.-Y., Su N.-W. (2018). Phosphorylation of isoflavones by *Bacillus subtilis* BCRC 80517 may represent xenobiotic metabolism. J. Agric. Food Chem..

[17] Smithberg M.H., Weiser C.J. (1968). Patterns of variation among climatic races of Red-Osier Dogwood. Ecology.

[18] Feild T.S., Lee D.W., Holbrook N.M. (2001). Why leaves turn red in autumn. The role of anthocyanins in senescing leaves of red-osier dogwood. Plant Physiol..

[19] Wei L.Y., Gomaa W.M.S., Ametaj B.N., Alexander T.W., Yang W.Z. (2019). Feeding red osier dogwood (*Corms sericea*) to beef heifers fed a high-grain diet affected feed intake and total tract digestibility. Anim. Feed Sci. Tech..

[20] Wei L.Y.Y., Jiao P.X.X., Alexander T.W., Yang W.Z. (2018). Inclusion of Red osier dogwood in high-forage and high-grain diets affected in vitro rumen fermentation. Ann. Anim. Sci..

[21] Koo B., Amarakoon S., Jayaraman B., Siow Y., Prashar S., Shang Y., Nyachoti C. (2018). Effects of dietary supplementation with ground red-osier dogwood (*Cornus stolonifera*) on oxidative status in weaned pigs challenged with *Escherichia coli* K88^+^. J. Anim. Sci..

[22] Jayaraman B., Amarakoon S., Koo B., O K., Nyachoti C. (2018). Effects of dietary supplementation with ground red-osier dogwood (*Cornus stolonifera*) on growth performance, blood profile, and ileal histomorphology in weaned pigs challenged with *Escherichia coli* K88^+^. J. Anim. Sci..

[23] Yang J., Zhu C.U.I., Ye J.l., Lv Y., Wang L., Chen Z., Jiang Z.y. (2019). Resveratrol protects porcine intestinal epithelial cells from deoxynivalenol induced damage via the Nrf2 signaling pathway. J. Agric. Food Chem..

[24] Wu H., Luo T., Li Y.M., Gao Z.P., Zhang K.Q., Song J.Y., Xiao J.S., Cao Y.P. (2018). Granny Smith apple procyanidin extract upregulates tight junction protein expression and modulates oxidative stress and inflammation in lipopolysaccharide-induced Caco-2 cells. Food Funct..

[25] Tian T., Zhang J., Wang Z. (2017). Pathomechanisms of oxidative stress in inflammatory bowel disease and potential antioxidant therapies. Oxid. Med. Cell. Longev..

[26] Wu J., Hillier C., Komenda P., Faria R.L.D., Santos S., Levin D., Zhang M., Lin F. (2016). An all-on-chip method for testing neutrophil chemotaxis induced by fMLP and COPD patient’s sputum. Technology.

[27] Serra G., Incani A., Serreli G., Porru L., Melis M.P., Tuberoso C.I.G., Rossin D., Biasi F., Deiana M. (2018). Olive oil polyphenols reduce oxysterols -induced redox imbalance and pro-inflammatory response in intestinal cells. Redox Biol..

[28] Tao L., Forester S.C., Lambert J.D. (2014). The role of the mitochondrial oxidative stress in the cytotoxic effects of the green tea catechin, (-)-epigallocatechin-3-gallate, in oral cells. Mol. Nutr. Food Res..

[29] Sakuma S., Abe M., Kohda T., Fujimoto Y. (2015). Hydrogen peroxide generated by xanthine/xanthine oxidase system represses the proliferation of colorectal cancer cell line Caco-2. J. Clin. Biochem. Nutr..

[30] Wang X., Zhao Y., Yao Y., Xu M., Du H., Zhang M., Tu Y. (2017). Anti-inflammatory activity of di-peptides derived from ovotransferrin by simulated peptide-cut in TNF-α-induced Caco-2 cells. J. Funct. Food..

[31] Mahmoud A.M., Germoush M.O., Al-Anazi K.M., Mahmoud A.H., Farah M.A., Allam A.A. (2018). Commiphora molmol protects against methotrexate-induced nephrotoxicity by up-regulating Nrf2/ARE/HO-1 signaling. Biomed. Pharmacother..

[32] Isaak C.K., Petkau J.C., Karmin O., Ominski K., Rodriguez-Lecompte J.C., Siow Y.L. (2013). Seasonal variations in phenolic compounds and antioxidant capacity of *Cornus stolonifera* plant material: Applications in agriculture. Can. J. Plant Sci..

[33] Yu L., Liu S., Yang C., O K., Adewole D., Sid V., Wang B. (2019). Rutin attenuates inflammatory responses induced by lipopolysaccharide in an in vitro mouse muscle cell (C2C12) model. Poultry Sci..

[34] Aggarwal S., Suzuki T., Taylor W.L., Bhargava A., Rao R.K. (2011). Contrasting effects of ERK on tight junction integrity in differentiated and under-differentiated Caco-2 cell monolayers. Biochem. J..

[35] Wijeratne S.S.K., Cuppett S.L., Schlegel V. (2005). Hydrogen peroxide induced oxidative stress damage and antioxidant enzyme response in Caco-2 human colon cells. J. Agric. Food Chem..

[36] Shin W., Kim H.J. (2018). Intestinal barrier dysfunction orchestrates the onset of inflammatory host–microbiome cross-talk in a human gut inflammation-on-a-chip. Proc. Natl. Acad. Sci. USA.

[37] Vreeburg R.A., van Wezel E.E., Ocaña-Calahorro F., Mes J.J. (2012). Apple extract induces increased epithelial resistance and claudin 4 expression in Caco-2 cells. J. Sci. Food Agric..

[38] Suzuki T., Hara H. (2009). Quercetin enhances intestinal barrier function through the assembly of zonnula occludens-2, occludin, and claudin-1 and the expression of claudin-4 in Caco-2 cells. J. Nutr..

